# Haplowebs as a graphical tool for delimiting species: a revival of Doyle's "field for recombination" approach and its application to the coral genus *Pocillopora *in Clipperton

**DOI:** 10.1186/1471-2148-10-372

**Published:** 2010-11-30

**Authors:** Jean-François Flot, Arnaud Couloux, Simon Tillier

**Affiliations:** 1 Courant Research Center "Geobiology", University of Göttingen, Goldschmidtstr. 3, 37077 Göttingen, Germany; 2 GENOSCOPE, Centre National de Séquençage, 2 rue Gaston Crémieux, CP5706, 91057 Evry Cedex, France; 3 UMR UPMC-CNRS-MNHN-IRD 7138, Département Systématique et Évolution, Muséum National d'Histoire Naturelle, Case Postale 26, 57 rue Cuvier, 75231 Paris Cedex 05, France; 4 URBO, Department of Biology, University of Namur, Rue de Bruxelles 61, 5000 Namur, Belgium

## Abstract

**Background:**

Usual methods for inferring species boundaries from molecular sequence data rely either on gene trees or on population genetic analyses. Another way of delimiting species, based on a view of species as "fields for recombination" (FFRs) characterized by mutual allelic exclusivity, was suggested in 1995 by Doyle. Here we propose to use haplowebs (haplotype networks with additional connections between haplotypes found co-occurring in heterozygous individuals) to visualize and delineate single-locus FFRs (sl-FFRs). Furthermore, we introduce a method to quantify the reliability of putative species boundaries according to the number of independent markers that support them, and illustrate this approach with a case study of taxonomically difficult corals of the genus *Pocillopora *collected around Clipperton Island (far eastern Pacific).

**Results:**

One haploweb built from intron sequences of the ATP synthase β subunit gene revealed the presence of two sl-FFRs among our 74 coral samples, whereas a second one built from ITS sequences turned out to be composed of four sl-FFRs. As a third independent marker, we performed a combined analysis of two regions of the mitochondrial genome: since haplowebs are not suited to analyze non-recombining markers, individuals were sorted into four haplogroups according to their mitochondrial sequences. Among all possible bipartitions of our set of samples, thirteen were supported by at least one molecular dataset, none by two and only one by all three datasets: this congruent pattern obtained from independent nuclear and mitochondrial markers indicates that two species of *Pocillopora *are present in Clipperton.

**Conclusions:**

Our approach builds on Doyle's method and extends it by introducing an intuitive, user-friendly graphical representation and by proposing a conceptual framework to analyze and quantify the congruence between sl-FFRs obtained from several independent markers. Like delineation methods based on population-level statistical approaches, our method can distinguish closely-related species that have not yet reached reciprocal monophyly at most or all of their loci; like tree-based approaches, it can yield meaningful conclusions using a number of independent markers as low as three. Future efforts will aim to develop programs that speed up the construction of haplowebs from FASTA sequence alignments and help perform the congruence analysis outlined in this article.

## Background

Species delimitation is an old issue in biology that continues to attract considerable attention [1-3] as the present global biodiversity crisis makes it of paramount importance to delineate and identify as objectively as possible species-level taxa [4]. The widespread occurrence of cryptic species [5] and the problems they pose in ecological, physiological and genetic studies [6] also call for the establishment of methods that can be applied by scientists from a variety of backgrounds and not solely by specialized taxonomists.

As pointed out by de Queiroz [7], diverging lineages acquire progressively through time a number of different properties that allow their recognition as distinct species. Among the different categories of characters (e.g., morphological, immunological, ecological or molecular) suitable for assessing the divergence between lineages, DNA sequences have gained increasing popularity among taxonomists in recent years as they are, in many cases, not influenced by environmental conditions nor by the life stage of the organisms under scrutiny; moreover, gathering information on DNA markers requires less taxon-specific training and expertise than for other categories of characters [8]. Methods for delimiting species based on molecular sequence data can be broadly classified in two categories: tree-based, and non-tree-based [1]. Tree-based methods use models of sequence evolution to reconstruct nodes that represent hierarchical kinship relationships among organisms in a historical perspective, whereas non-tree-based approaches use population genetic models to look for evidence of barriers or restrictions to gene flow among and within extant populations.

**Figure 1:**
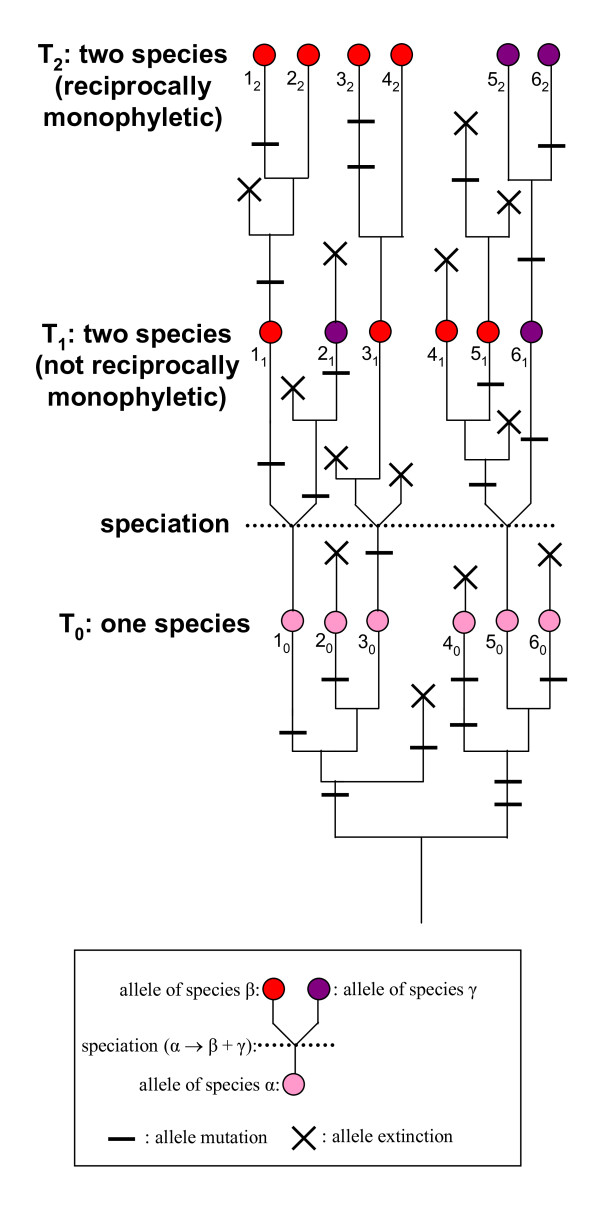
**Mutual exclusivity vs. reciprocal monophyly**. To illustrate the concepts of mutual exclusivity and reciprocal monophyly, let us visualize how the alleles in a gene tree are distributed at the various stages of the process of speciation. Unless the genetic polymorphism of the ancestor species is very low (for instance following a strong bottleneck event), sequencing at T_0 _any variable marker from a number of individuals of this species would yield a diversity of sequences (haplotypes 1_0 _to 6_0_, in pink). Following speciation, the two resulting sister species inherit the polymorphism of their common ancestor and are thus initially indistinguishable, but their sequences immediately start to diverge as some haplotype lineages get extinct through genetic drift (lineage sorting) while others accumulate mutations. If effective population sizes are large, genetic drift acts more slowly than mutations: in such case, the two sister species become genetically distinguishable at T_1 _when their sets of sequences are mutually exclusive (haplotypes 1_1_, 3_1_, 4_1_, 5_1 _in red, haplotypes 2_1 _and 6_1 _in blue), i.e. the two species do not share any sequence; reciprocal monophyly is reached at a later stage (T_2_, haplotypes 1_2 _to 6_2_), or may theoretically never be reached if the effective population size is infinite. In all cases, mutual exclusivity is reached before or at the same time as reciprocal monophyly: hence, mutual exclusivity is a more powerful and sensitive criterion than reciprocal monophyly to delineate species.

Even though "a highly corroborated hypothesis of lineage separation (i.e., of the existence of separate species) requires multiple lines of evidence" [9], as a first approach it may be desirable to choose a species delimitation criterion that is as general as possible and can be used systematically. Since a recent survey found 23% of species reported in the literature to be either poly- or paraphyletic at the various loci investigated [10], a sensitive delimitation method should be capable of detecting closely related species at an early stage of lineage divergence, when most of their genetic loci have not yet reached reciprocal monophyly (Figure 1). Moreover, it would be advantageous to use as a general yardstick for delimiting species a method that does not make restrictive assumptions regarding the genetics of the marker used (e.g., the absence of copy-number variations) and of the populations studied (e.g., random mating).

One such method applicable to sexually reproducing organisms was proposed 15 years ago by Doyle [11]: briefly, this approach uses information on the co-occurrence of alleles in the diploid phase to delineate, for each marker investigated, groups of individuals sharing a common "allele pool". In Doyle's terminology these groups of individuals are named "fields for recombination" (FFRs), following an earlier proposal by Carson [12] to consider species as groups of individuals whose alleles recombine through segregation and meiosis. Doyle's method relies on the expectation that it takes less time for diverging populations to reach mutual allelic exclusivity than reciprocal allelic monophyly (Figure 1): hence, groups of individuals that do not have any allele in common can be assumed to belong to distinct species even though they are not reciprocally monophyletic.

In Doyle's original proposal, a first step is to delineate, for each genetic locus under scrutiny, single-locus FFRs (sl-FFRs). However, "with sufficiently fine resolution, the allele pool may not extend beyond the single heterozygous individuals in which two alleles coexist": in such situation, "many more allele pools are recognized, and concomitantly there are more FFRs, each consisting of a single individual" [11]. To overcome this problem, Doyle proposes to use information on the co-occurrence of alleles from different loci to lump sl-FFRs into multilocus FFRs (ml-FFRs): if individuals A and B belong to the same sl-FFR for marker 1, and individuals B and C belong to the same ssl-FFR for marker 2, then individuals A, B and C belong to the same ml-FFR. One drawback of this approach, however, is that it combines information from all available markers, which makes it difficult to judge the reliability of the ml-FFRs obtained (for instance, the inclusion of a single ancestrally shared or recently introgressed sequence in a dataset would cause the whole analysis to yield incorrect conclusions). Moreover, Doyle's method requires determining the alleles of many individuals for several codominant nuclear markers: until recently, this could only be done for low-resolution and/or homoplasy-plagued markers such as allozymes or microsatellites, which probably explains why earlier attempts to use this method for species delimitation were unsuccessful [13-15].

In the last few years, new techniques have emerged that make it possible to obtain information-rich allelic sequences from heterozygous individuals without cloning [16,17]. Sequences obtained from exon-primed, introns-crossing (EPIC) nuclear markers [18], as a result, appear now well suited for delimiting species using Doyle's method. And since such sequences are of finite (and usually moderate) length, a simple strategy to obtain sl-FFRs that accurately delineate reproductively isolated populations is to sequence a large number of individuals. One may then assess the reliability of the resulting species boundaries by checking whether they are supported by several independent molecular markers, a congruence approach [19,20] that presents the advantage of not mixing together information from different sources.

**Figure 2:**
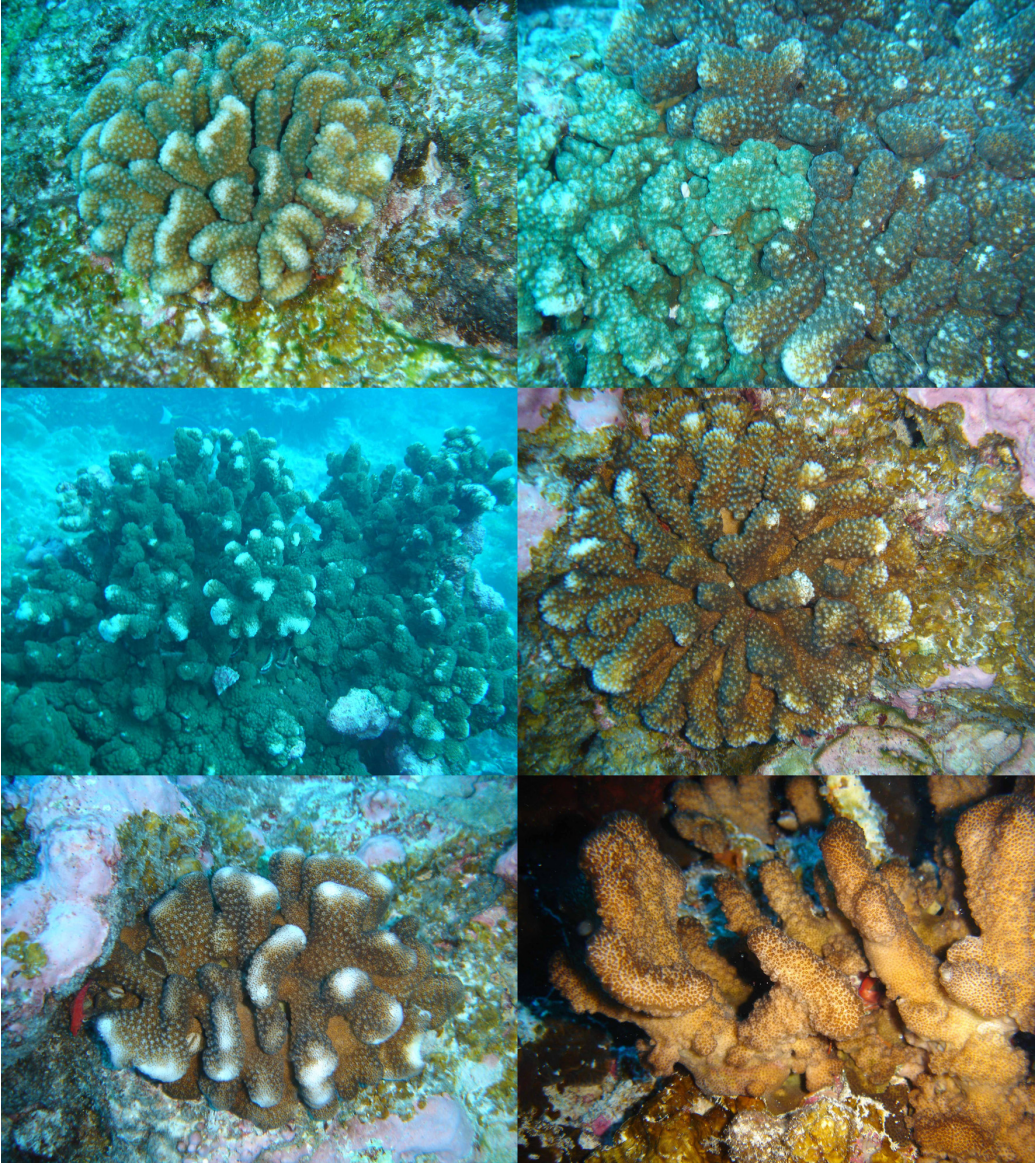
**Morphological diversity of the coral genus *Pocillopora *in Clipperton**. Corals of the genus *Pocillopora *are common on nearly all tropical reefs except in the Caribbean. Their taxonomy is extremely confusing due to their extensive phenotypic plasticity, and clear-cut diagnostic morphological characters are missing for most currently defined species [35]: hence, individuals that cannot be reliably identified are common. Clipperton Island is a very good and somewhat extreme example of this confusion, with different taxonomic experts having recognized successively three [50], one or two [51], three [52] and six [53] species among specimens collected around this atoll. We illustrate here a small sample of this morphological variation: from left to right and top to bottom, colony 05Clip026, colonies 05Clip052 (left side of the picture, green) and 05Clip053 (right side of the picture, brown), colony 05Clip045, colony 05Clip018, colony 05Clip019, colony 05Clip002. According to the results of the molecular analyses presented in this article, the colonies in the four first photographs belong to one species (*Pocillopora *sp. A) and the last two colonies belong to another (*Pocillopora *sp. B), a delineation far from obvious based on their morphology.

Here we propose to revive and invigorate Doyle's approach by extending it in two directions. First, we present a reliable graphical method to delineate sl-FFRs in large datasets using haplowebs (a contraction of "haplotype webs"): these two-dimensional representations are obtained from conventional haplotype networks ("haplonets") by adding connections between haplotypes found co-occurring in heterozygous individuals, i.e. haplotypes that belong to the same allele pool *sensu *Doyle. And second, we introduce a procedure to complement the ml-FFR approach (used by Doyle to infer species boundaries from several markers) with an analysis of the congruence between markers, by scoring each possible bipartition of the set of individuals according to the number of independent marker supporting it. These two approaches are illustrated with a detailed example of their application to taxonomically difficult corals of the genus *Pocillopora *(Figure 2) collected around Clipperton, an atoll located in the far eastern Pacific Ocean.

## Results

### Nuclear and mitochondrial markers yield putative species-level groupings of individuals

Two nuclear markers and two fragments of the mitochondrial genome were successfully sequenced from each of the 74 *Pocillopora *samples that we had collected around Clipperton. Haplonets were constructed for each nuclear marker and for the concatenation of the two mitochondrial markers; the haplonets obtained from each nuclear marker were subsequently converted into haplowebs by drawing additional connections between haplotypes found co-occurring in heterozygous individuals.

**Figure 3:**
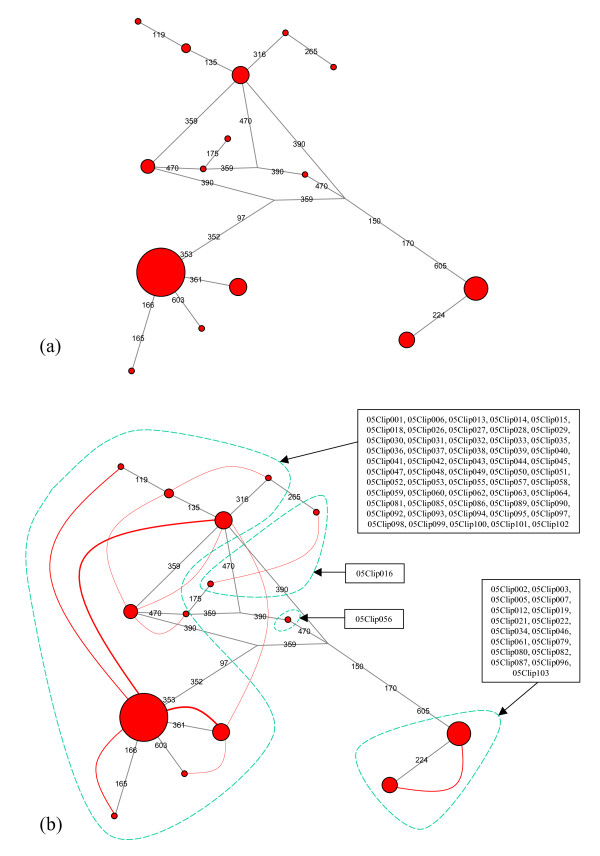
**ITS haplonet and haploweb**. (a) As a first step in the analysis, a haplotype network (in short, "haplonet") was build from the alignment of all ITS sequences. Since 31 individuals were heterozygous and 43 were homozygous for this marker, the alignment comprised 105 sequences, among which 15 different haplotypes could be distinguished (represented as circles on the haplonet, with diameters proportional to the number of individuals harboring each of them). Each haplotype is connected to one or several others by straight lines representing the evolutionary paths inferred by the network-building algorithm (numbers in red on the lines represent mutated positions in the alignment). (b) As a second step, the haplonet was converted into a haplotype web (in short, "haploweb") by adding curves connecting haplotypes found co-occurring in heterozygous individuals (the width of each curve is drawn proportional to the number of heterozygotes harboring the two haplotypes it connects). This allowed us to delineate 4 pools of co-occurring haplotypes (enclosed in green dashes on the figure). To each of these 4 allele pools corresponds a group of individuals (called single-locus fields for recombination, or sl-FFR in Doyle's terminology), whose names are listed inside boxes with arrows pointing on the corresponding allele pool: these 4 sl-FFRs comprised respectively 55, 17, 1 and 1 individuals.

The internal transcribed spacers (ITS) are variable non-coding regions located in the nuclear ribosomal DNA that have been proposed as a universal species-level marker in corals [21]. In the present study we focused on the ITS2 region, located between the 5.8S and 28S ribosomal genes. Fifteen different ITS2 sequences were detected (Figure 3a), the most common of which was found in 47 individuals (out of 74) whereas eight sequences were singletons (i.e., were only present in one individual each). Visual inspection of the resulting haploweb (Figure 3b) revealed four allele pools (*sensu *Doyle) comprising 10, 2, 2 and 1 sequences; the corresponding four sl-FFRs comprised 55, 17, 1 and 1 individuals, respectively.

**Figure 4:**
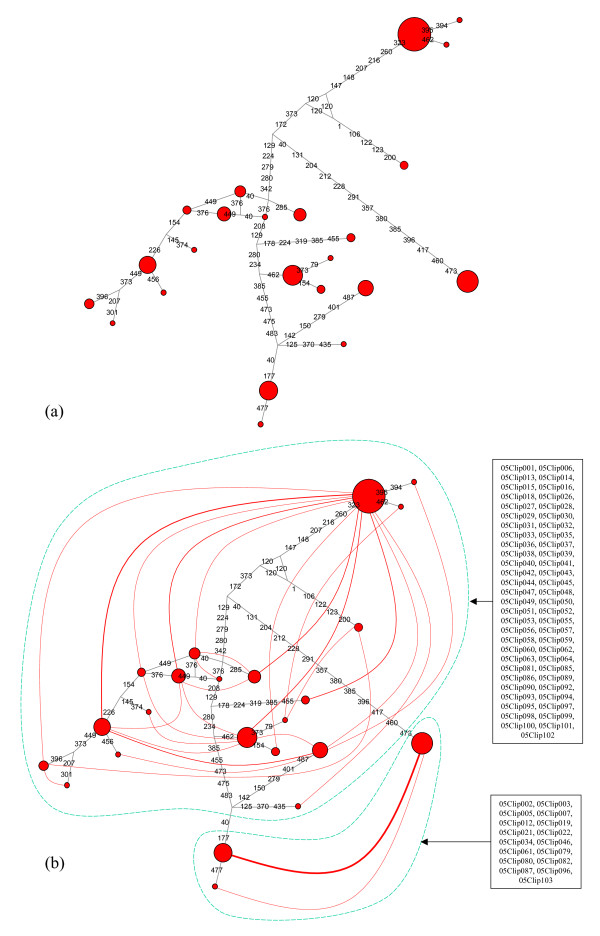
**ATPSβ haplonet and haploweb**. (a) For the ATPSβ marker there were 28 homozygotes and 46 heterozygotes: hence, the alignment comprised 120 sequences, among which 25 distinct haplotypes could be distinguished. (b) There were 2 pools of co-occurring haplotypes (enclosed in green dashes on the figure) in the ATPSβ haploweb, corresponding to 2 sl-FFRs that comprised respectively 17 and 57 individuals.

As a second supposedly independent nuclear marker, we sequenced an intron of the ATP synthase β subunit (ATPSβ) gene: 23 distinct ATPSβ haplotypes were detected (Figure 4), the most common of which was found in 32 individuals whereas 9 haplotypes were singletons. The corresponding haploweb revealed two sl-FFRs: the larger allele pool included 20 haplotypes found in 57 individuals, whereas the smaller one included 3 haplotypes found in 17 individuals.

**Figure 5:**
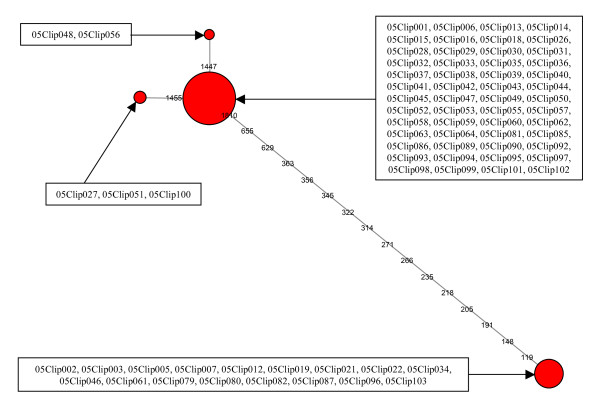
**Mitochondrial haplonet**. Since there was only one mitochondrial sequence per individual, the haplonet obtained from this marker could not be converted into a haploweb (there were no co-occurring haplotypes). However, 4 haplogroups could be distinguished that comprised respectively 52, 17, 3 and 2 individuals.

As for the two mitochondrial fragments (the putative control region and a highly variable ORF of unknown function [22]), haplowebs were not suited to analyze them since they were not recombining (a single haplotype was found in each individual, as is usually the case with mitochondrial markers); hence, individuals were simply sorted into haplogroups according to the mitochondrial haplotype they possessed. Only four different haplotypes were detected among the 74 samples analyzed (Figure 5): the most common haplotype, found in 52 coral colonies, was separated by only one mutation from the two less common ones (respectively found in 2 and 3 individuals), whereas the fourth haplotype, present in 17 individuals, was 16 mutations away.

### The congruence between independent groupings can be quantified using bipartition scores

Each of our three independent datasets (ITS, ATPSβ and the combined mitochondrial regions) supported several possible species boundaries, but combining the results of the two nuclear markers following Doyle's approach yielded two ml-FFRs comprising respectively 57 and 17 individuals. To assess the reliability of this result, we considered all possible bipartitions of our 74 samples and scored them according to the number of markers supporting them (Table 1); the mitochondrial regions were included in this analysis as a single marker independent from the two nuclear ones. Generally speaking, the number of possible bipartitions of a set of n objects is 2^n-1^-1 (if one excludes the trivial case "all objects vs. none of them"): hence, a dataset comprised of two sl-FFRs (or two haplogroups) supports a single bipartition, a dataset comprised of three sl-FFRs (or three haplogroups) supports 3 bipartitions, a dataset comprised of four sl-FFRs (or four haplogroups) supports 7 bipartitions, etc.

**Table 1:** Bipartitions of our set of samples and the molecular markers supporting them

	**Bipartitions**	**ITS2**	**ATPSβ**	**ORF+CR**	**support**
	
1	G vs. all other samples	√	√	√	100%
	
2	05Clip016 vs all others	√			33%
	
3	05Clip056 vs. all others	√			33%
	
4	05Clip016+05Clip056 vs. all others	√			33%
	
5	G+05Clip016 vs. all others	√			33%
	
6	G+05Clip056 vs. all others	√			33%
	
7	G+05Clip016+05Clip 056 vs. all others	√			33%
	
8	05Clip048+05Clip056 vs. all others			√	33%
	
9	05Clip027+05Clip051+05Clip100 vs. all others			√	33%
	
10	05Clip048+05Clip027+05Clip056+05Clip051+05Clip100 vs. all others			√	33%
	
11	G+05Clip048+05Clip056 vs. all others			√	33%
	
12	G+05Clip027+05Clip051+05Clip100 vs. all others			√	33%
	
13	G+05Clip048+05Clip027+05Clip056+05Clip051+05Clip100 vs. all others			√	33%

G = 05Clip002+05Clip003+05Clip005+05Clip007+05Clip012+05Clip019+05Clip021+05Clip022+05Clip034 +05Clip046+05Clip061+05Clip079+05Clip080+05Clip082+05Clip087+05Clip096+05Clip103

The ATPSβ haploweb detected a single putative species boundary in our dataset, partitioning it in two sl-FFRs comprising respectively 57 and 17 individuals. Since the ITS2 haploweb was comprised of 4 sl-FFRs, it supported 7 possible bipartitions of our set of 74 individuals, only one among which was also supported by the other nuclear marker. As for the combined mitochondrial regions, they delineated four haplogroups among our samples (as defined by the mitochondrial haplotype they harbored): out of the 7 possible bipartitions supported by this marker, one was also supported by the ITS2 and ATPSβ datasets.

**Figure 6:**
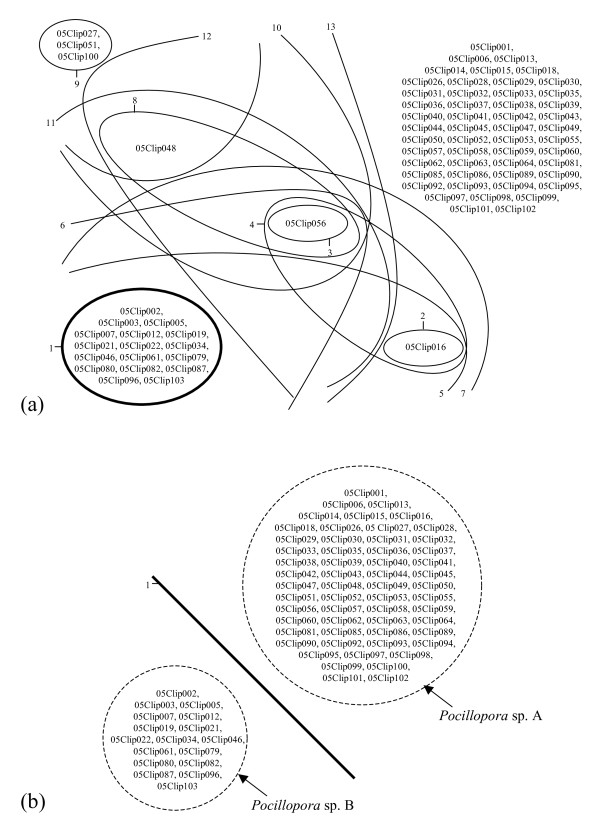
**Bipartition reconciliation**. (a) In order to determine graphically the number of well-supported groups of individuals, bipartitions can be represented on a two-dimensional figure as lines or curves (each of them dividing the group of samples analyzed into two). Some groups of individuals observable in such graph do not correspond to sl-FFRs but rather to intersections of sl-FFRs obtained from different markers: here, this is the case of individual 05Clip048 and of the large group of samples in the upper right corner of the figure, whose support is equal to zero as these groups are not supported by any single marker. Each bipartition is numbered from 1 to 13 to facilitate comparison with the respective lines of Table 1, and is drawn with its thickness proportional to the number of independent datasets supporting it. (b) Bipartitions below an arbitrary support threshold (here, 50%) may be omitted from such graph for the sake of clarity.

Among the 13 bipartitions supported by at least one marker, none was supported by two markers and one was supported by all of them (Table 1). This single well-supported bipartition divides our set of samples into two populations of 57 and 17 individuals (Figure 6) that do not share any allele at the three independent genetic loci investigated: according to the mutual allelic exclusivity criterion (Figure 1), these two populations thus represent distinct species, here designated *Pocillopora *sp. A and *Pocillopora *sp. B pending further taxonomic examination.

## Discussion

### Using haplowebs to delineate sl-FFRs: delimiting species without *a priori *hypotheses

As illustrated with our *Pocillopora *example, haplowebs provide a simple, intuitive way to delineate sl-FFRs from a set of nuclear sequences and represent them graphically. In this article we chose to represent haplowebs by adding connections between co-occurring haplotypes on top of haplotype networks (since network-building algorithms are arguably more suited to reconstruct intra-specific genealogies than are phylogenetic methods [23]), but it should be emphasized that haplowebs can also be drawn atop phylogenetic trees if one wishes to do so.

Usual tree-based methods can only delineate species that are reciprocally monophyletic (except for some species-tree approaches based on the coalescent that show great promise for the future [24] but still often return incorrect results when applied to species delimitation [25]), whereas most non-tree-based methods are statistical in nature and require large sample sizes and numbers of markers in order for meaningful conclusions to be reached. Another issue with such statistical approaches is that genotypes have to be determined for each individual sampled, which poses problems in cases of polyploïdy, aneuploidy, copy-number variation or chimerism when individuals comprise variable numbers of haplotypes and the genotypes cannot be determined (as we experienced in a previous study of *Pocillopora *corals from Hawaii [26]). In contrast, the delineation of allele pools using Doyle's method is based only on haplotype presence/absence information and thus does not require knowledge of the respective amount of each haplotype in the genotype.

Even though haplowebs have their most obvious application in the analysis of nuclear sequence markers, one may also find them useful when attempting to delineate species using mitochondrial markers that co-amplify with nuclear pseudogenes (Numts [27]). Such pseudogenes are common in many taxa [28-32] and can be very difficult to tell apart from *bona fide *mitochondrial sequences [33]. However, pseudogene sequences diverge following speciation just like other markers, and reproductively isolated species are thus expected to own mutually exclusive sets of paralogous sequences that may not be reciprocally monophyletic but will be easily detected using haplowebs.

A previous survey of *Pocillopora*'s molecular diversity based on a single nuclear marker (ITS2) also included four samples from Clipperton [34]: in this survey, however, the authors took the morphological delimitation of species in Veron's *Corals of the World *[35] as granted and thus attributed all their samples from Clipperton to the species "*Pocillopora effusus*". Even though they presented compelling evidence for the presence of three divergent ITS2 types among their samples, they did not envision in their article the possibility that cryptic *Pocillopora *species may be present but rather decided to attribute the observed molecular diversity to interspecific hybridization. This highlights that molecular studies based on a single marker can easily yield erroneous conclusions, especially when *a priori *morphological hypotheses hinder the objective analysis of the molecular data at hand. Moreover, this suggests that the current evidence for interspecific hybridization in corals will have to be carefully reevaluated once their species-level taxonomy becomes clarified.

### Bipartition scoring vs. Doyle's ml-FFR approach

If analyses using different markers yield different sl-FFRs, then the simplest explanation is that not enough individuals were sequenced, resulting in some sl-FFRs that are artefactually smaller than in reality. A straightforward way of solving this discrepancy would be to increase the number of individuals in the dataset, but this cannot always be done: for instance, the sampled populations may be rare or endangered, the sampling site may be difficult to access, or time and money may be limiting factors. A good example of the consequences of severe undersampling can be found in our previously published study of the genus *Pocillopora *in Hawaii [26], in which a first attempt to delineate graphically sl-FFRs was presented: for each of the four nuclear markers analyzed, numerous small sl-FFRs were obtained as the number of individuals sampled (37 in total) turned out to be very insufficient. In less severe cases, however, Doyle's ml-FFR method and/or the bipartition scoring approach presented here can be used to synthesize the results obtained from all markers and obtain a putative species delimitation based on all information available at hand.

Although Doyle's ml-FFR procedure may superficially be mistaken for an approach based on congruence (as it starts first by analyzing each marker separately to find out sl-FFRs, then combines all the results), it is better described as a "total evidence" [36,37] approach since the inclusion of a single aberrant dataset can cause the whole analysis to yield conclusions that are not supported by any other marker. A possible way to detect and eliminate such aberrant marker could be to remove one locus at a time and delineate ml-FFRs using the remaining ones, but such approach is very time-consuming and will perform poorly if there are several aberrant datasets. Our bipartition-scoring approach, however, solves this problem by making it possible to compare and quantify the support brought by each marker to the putative species boundaries.

Missing data, when extensive, can jeopardize phylogenetic analyses [38,39], but Doyle's ml-FFR approach is relatively immune to this problem since possession of a single sequence from a known allele pool is a sufficient criterion for attributing an individual to a given ml-FFR (even when the sequences of this individual for all other markers are unavailable). The bipartition scoring approach presented here, however, only works if sl-FFRs for all markers are delineated from the same set of individuals.

Whereas the ml-FFR method proposed by Doyle only bases itself on the sl-FFRs obtained from nuclear markers, our bipartition scoring approach can include other types of groupings based on a variety of characters: haploid sequence markers (such as the mitochondrial regions used in our *Pocillopora *example), morphological characters, biochemical or immunological properties, behavior, etc. Including a few morphological, biochemical or behavioral characters in the bipartition scoring analysis would provide a nice way to test the congruence of the patterns obtained from there characters with those obtained from molecular sequence markers. However, our method of quantifying support supposes that the grouping used (i.e., all columns in Table 1) be independent from each other, a requirement easily testable in the case of molecular markers (since non-independent markers are expected to yield congruent gene trees) but that may prove more difficult to establish for other kinds of characters: hence, one may prefer to use solely molecular characters for quantifying the support of the bipartitions.

### Possible pitfalls: dealing with selection, shared ancestral sequences and introgression/hybridization

One possible issue with using the criterion of mutual allelic exclusivity to delineate species is that populations inhabiting contrasting environment can have distinct alleles at loci that are differentially selected: for such markers under selection, one may then end up with sl-FFRs that are less encompassing than the real species as they rather delineate intra-specific ecological niches. However, this problem will only affect markers that are selected: if several markers are used, there is good chance that most of them will be neutral or near-neutral (or that they will be subjected to different selective pressures), in which case the congruence analysis presented here will still recover the true species boundaries. Moreover, whenever two sl-FFRs yielded by a marker turn out to be sympatric populations inhabiting the same environment, then the chance that the putative species boundary between them be actually an artefact caused by selection becomes vanishingly small.

Recent introgression and shared ancestral sequences are two other possible pitfalls of Doyle's ml-FFR method: the inclusion of a single recently introgressed sequence or shared ancestral haplotype in a dataset supporting otherwise the delimitation of species A and B would cause the immediate collapse of the two corresponding ml-FFRs into a single unit and yield the erroneous conclusion that A and B are conspecific. However, the bipartition scoring approach presented here would not be affected by the inclusion of a few such "misbehaving" loci as long as a majority of the markers do behave properly. F1 hybrids present a more difficult problem since they cannot be detected by a congruence analysis such as our bipartition scoring approach. However, if F1 hybrids are relatively rare in the population (as is usually the case for interspecific hybrids), then they may be spotted in haplowebs as thin lines connecting large haplotype circles (i.e., infrequent co-occurrence of common haplotypes, which runs contrary to random-mating expectations). If the same small set of individuals turns out to be responsible for such infrequent connections over nearly all molecular markers analyzed, one may assume with a high level of certainty that these are F1 hybrids and subsequently remove them from the analysis to ensure proper species delimitation.

## Conclusions

Haplowebs are versatile tools that combine properties from both tree-based and non-tree-based approaches to species delineation: like the former, haplowebs can provide meaningful conclusions from relatively few markers without relying on population genetic models, and like the latter, they allow detection of potential species boundaries at an early stage of divergence when populations have not yet reached reciprocal allelic monophyly. The method used for building haplowebs from sets of sequences and for analyzing the congruence between them is straightforward and reproducible: hence, our next step will be to develop programs that speed up the construction of haplowebs from FASTA sequence alignments and help perform the congruence analysis presented in this article.

## Methods

### Sample collection and processing

Fragments from 74 *Pocillopora *coral colonies were collected while scuba diving or snorkeling on the reefs surrounding Clipperton Island (10°18'00''N, 109°13'00"W) from 5 to 14 March 2005 during the international expedition organized by Jean-Louis Etienne. Each colony sampled was photographed underwater and its depth recorded (Table 2). Coral tissues were preserved in buffered guanidium thiocyanate solution [40,41] and their DNA purified on an ABI Prism 6100 Nucleic Acid PrepStation.

**Table 2:** Localization and depth of each *Pocillopora* sample collected in Clipperton

Sample name	Coordinates	Depth (m)		Sample name	Coordinates	Depth (m)
05Clip001	(10°17'32"N, 109°13'34"W)	30.0		05Clip048	(10°17'07"N, 109°12'35"W)	11.0

05Clip002	(10°17'32"N, 109°13'34"W)	26.0		05Clip049	(10°17'07"N, 109°12'35"W)	11.0

05Clip003	(10°17'32"N, 109°13'34"W)	25.5		05Clip050	(10°17'07"N, 109°12'35"W)	11.0

05Clip005	(10°17'32"N, 109°13'34"W)	21.3		05Clip051	(10°17'07"N, 109°12'35"W)	11.0

05Clip006	(10°17'32"N, 109°13'34"W)	15.5		05Clip052	(10°17'07"N, 109°12'35"W)	11.0

05Clip007	(10°17'32"N, 109°13'34"W)	15.0		05Clip053	(10°17'07"N, 109°12'35"W)	11.0

05Clip012	(10°17'38"N, 109°13'50"W)	10.0		05Clip055	(10°18'51"N, 109°14'16"W)	30.0

05Clip013	(10°17'38"N, 109°13'50"W)	10.0		05Clip056	(10°18'51"N, 109°14'16"W)	24.3

05Clip014	(10°17'38"N, 109°13'50"W)	10.0		05Clip057	(10°18'51"N, 109°14'16"W)	24.1

05Clip015	(10°17'38"N, 109°13'50"W)	10.0		05Clip058	(10°18'51"N, 109°14'16"W)	22.0

05Clip016	(10°17'38"N, 109°13'50"W)	10.0		05Clip059	(10°18'51"N, 109°14'16"W)	19.2

05Clip018	(10°17'38"N, 109°13'50"W)	12.0		05Clip060	(10°18'51"N, 109°14'16"W)	17.5

05Clip019	(10°17'38"N, 109°13'50"W)	12.0		05Clip061	(10°18'51"N, 109°14'16"W)	25.6

05Clip021	(10°17'38"N, 109°13'50"W)	12.0		05Clip062	(10°18'51"N, 109°14'16"W)	19.7

05Clip022	(10°17'38"N, 109°13'50"W)	12.0		05Clip063	(10°18'51"N, 109°14'16"W)	17.0

05Clip026	(10°17'07"N, 109°12'35"W)	10.0		05Clip064	(10°18'51"N, 109°14'16"W)	15.5

05Clip027	(10°17'07"N, 109°12'35"W)	10.0		05Clip079	(10°18'51"N, 109°14'16"W)	24.2

05Clip028	(10°17'07"N, 109°12'35"W)	10.0		05Clip080	(10°18'51"N, 109°14'16"W)	22.1

05Clip029	(10°17'07"N, 109°12'35"W)	10.0		05Clip081	(10°18'51"N, 109°14'16"W)	22.2

05Clip030	(10°17'07"N, 109°12'35"W)	10.0		05Clip082	(10°18'51"N, 109°14'16"W)	21.9

05Clip031	(10°17'07"N, 109°12'35"W)	10.0		05Clip085	(10°18'51"N, 109°14'16"W)	18.3

05Clip032	(10°17'07"N, 109°12'35"W)	10.0		05Clip086	(10°18'51"N, 109°14'16"W)	17.2

05Clip033	(10°17'07"N, 109°12'35"W)	10.0		05Clip087	(10°18'51"N, 109°14'16"W)	15.0

05Clip034	(10°17'07"N, 109°12'35"W)	10.0		05Clip089	(10°17'57"N, 109°13'50"W)	1.0

05Clip035	(10°17'07"N, 109°12'35"W)	10.0		05Clip090	(10°17'57"N, 109°13'50"W)	1.0

05Clip036	(10°17'07"N, 109°12'35"W)	10.0		05Clip092	(10°17'28"N, 109°13'17"W)	1.0

05Clip037	(10°17'07"N, 109°12'35"W)	10.0		05Clip093	(10°17'28"N, 109°13'17"W)	1.0

05Clip038	(10°17'07"N, 109°12'35"W)	10.0		05Clip094	(10°17'28"N, 109°13'17"W)	1.0

05Clip039	(10°17'07"N, 109°12'35"W)	10.0		05Clip095	(10°17'28"N, 109°13'17"W)	1.0

05Clip040	(10°17'07"N, 109°12'35"W)	11.0		05Clip096	(10°17'28"N, 109°13'17"W)	1.0

05Clip041	(10°17'07"N, 109°12'35"W)	11.0		05Clip097	(10°18'00"N, 109°13'53"W)	1.0

05Clip042	(10°17'07"N, 109°12'35"W)	11.0		05Clip098	(10°18'00"N, 109°13'53"W)	1.0

05Clip043	(10°17'07"N, 109°12'35"W)	11.0		05Clip099	(10°18'00"N, 109°13'53"W)	1.0

05Clip044	(10°17'07"N, 109°12'35"W)	11.0		05Clip100	(10°18'00"N, 109°13'53"W)	1.0

05Clip045	(10°17'07"N, 109°12'35"W)	11.0		05Clip101	(10°18'00"N, 109°13'53"W)	1.0

05Clip046	(10°17'07"N, 109°12'35"W)	11.0		05Clip102	(10°18'00"N, 109°13'53"W)	1.0

05Clip047	(10°17'07"N, 109°12'35"W)	11.0		05Clip103	(10°18'00"N, 109°13'53"W)	1.0

### PCR amplification and sequencing

We selectively amplified and sequenced two regions of the mitochondrial genome previously identified as the most variable in *Pocillopora *[22], and two nuclear markers that had been shown to yield useful information on species delimitations in this genus [42,26]. Briefly, amplifications were performed in 25 μl reaction mixes containing 1x Red Taq buffer, 264 μM dNTP, 5% DMSO, 0.3 μM PCR primers (Table 3), 0.3 units Red Taq (Sigma), and 10-50 ng DNA. PCR conditions comprised an initial denaturation step of 60 s at 94°C, followed by 40-50 cycles (30 s denaturation at 94°C, 30 s annealing at 53°C, 75 s elongation at 72°C) and a final 5-min elongation step at 72°C. PCR products were sequenced in both directions (see primers in Table 3), and sequences were assembled and cleaned using Sequencher 4 (Gene Codes).

**Table 3:** Primers used for PCR amplification and sequencing

Marker	Primer name	Purpose	Sequence	Reference
ITS2 (nuclear)	ITSc2-5	PCR + sequencing	5'-AGCCAGCTGCGATAAGTAGTG-3'	[42]

ITS2 (nuclear)	R28S1	PCR + sequencing	5'-GCTGCAATCCCAAACAACCC-3'	[42]

ATPSβ (nuclear)	ATPSβf5	PCR	5'-CCAAGGGTGGNAARATHGGT-3'	this article

ATPSβ (nuclear)	ATPSβr2	PCR + sequencing	5'-GGTTCGTTCATCTGACCATACAC-3'	[26]

ATPSβ (nuclear)	ATPSβf2	sequencing	5'-TGAAAGACAAGAGCTCCAAGGTA-3'	[26]

ATPSβ (nuclear)	ATPSβf4	sequencing	5'-GAGCTGGTGTTGGAAAGACTGT-3'	this article

ORF (mitochondrial)	FATP6.1	PCR + sequencing	5'-TTTGGGSATTCGTTTAGCAG-3'	[26]

ORF (mitochondrial)	RORF	PCR + sequencing	5'-SCCAATATGTTAAACASCATGTCA-3'	[26]

ORF (mitochondrial)	FORF	sequencing	5'-GTGCGCCAGCATTCTATTG-3'	this article

ORF (mitochondrial)	RORF2	sequencing	5'-TAGAATGCTGGCGCACATAA-3'	this article

CR (mitochondrial)	FNAD5.2deg	PCR + sequencing	5'-GCCYAGRGGTGTTGTTCAAT-3'	[26]

CR (mitochondrial)	RCOI3deg	PCR + sequencing	5'-CGCAGAAAGCTCBARTCGTA-3'	[54]

CR (mitochondrial)	FNC1	sequencing	5'-GGGGTGAGATGAAGAGGTGA-3'	this article

CR (mitochondrial)	RNC1	sequencing	5'-CGGGTGCCACTATGTTTTCT-3'	this article

### Determination of nuclear haplotypes

The ITS2 chromatograms obtained from 31 individuals and the ATPSβ chromatograms obtained from 46 individuals contained double peaks, indicating that each of these individuals possessed two sequence types. Finding out the sequence types was trivial for 11 pairs of ITS2 chromatograms that contained only one double peak. The ITS2 chromatogram of 15 individuals and the ATPSβ chromatogram of 32 individuals had numerous double peaks as expected in the case of length-variant heterozygotes [43,16], and their superposed sequences were directly deconvoluted using the program CHAMPURU [44] (available online at http://www.mnhn.fr/jfflot/champuru). The remaining chromatograms (of 5 individuals for ITS2 and of 14 individuals for ATPSβ) had a few double peaks and belonged to heterozygotes whose allelic sequences were of identical lengths: those were resolved statistically by reference to the rest of the dataset using SeqPHASE [45] (available online at http://www.mnhn.fr/jfflot/seqphase) and PHASE [46]. All in all there were 53 distinct multilocus genotypes among our 74 samples; 46 multilocus genotypes turned out to belong to *Pocillopora *sp. A and the 7 others to *Pocillopora *sp. B.

### Construction of haplonets and haplowebs

Sequences were aligned in MEGA4 [47] and deposited in public databases [GenBank:FR729101-FR729473]. DNA regions so variable that homology was uncertain were removed from the ITS2 and ATPSβ alignments. Alignments were converted to the Roehl format using DnaSP [48]; median-joining haplotype networks [49] were constructed using Network 4.1 (available online at http://www.fluxus-engineering.com/) and converted into enhanced metafiles (emf) using Network Publisher (Fluxus Technology). Finally, enhanced metafiles were imported in Microsoft PowerPoint to add colors and connections between co-occurring haplotypes (drawn with their thickness proportional to the number of individuals in which the said haplotypes were found co-occurring).

### Bipartition scoring and reconciliation

Each possible bipartition of our 74 samples into two complementary sets of individuals was scored according to the number of independent datasets supporting it. The support of each bipartition was calculated as the percentage of independent datasets that supported this bipartition. A cutoff value of 50% was arbitrarily set to discriminate well-supported bipartitions from those with little or no support, and the bipartitions were reconciled in a two-dimensional graph to determine the number of well-supported groups of individuals (i.e., putative species) among our samples.

## Authors' contributions

JFF devised the method, carried out fieldwork, DNA extractions and PCR amplifications, analyzed the results and drafted the manuscript. AC sequenced all PCR products. ST supervised the study and revised the manuscript. All authors read and approved the final manuscript.
